# Lewis and AB0 blood group-phenotypes in periodontitis, cardiovascular disease, obesity and stroke

**DOI:** 10.1038/s41598-019-42594-z

**Published:** 2019-04-18

**Authors:** C. Enevold, C. H. Nielsen, D. Molbo, R. Lund, K. Bendtzen, N. -E. Fiehn, P. Holmstrup

**Affiliations:** 1 Institute for Inflammation Research, Center for Rheumatology and Spine Diseases, Rigshospitalet, Copenhagen, Denmark; 2 University of Copenhagen, Faculty of Health and Medical Sciences, Department of Odontology, Copenhagen, Denmark; 3 University of Copenhagen, Faculty of Health and Medical Sciences, Department of Public Health, Section of Social Medicine, Copenhagen, Denmark; 4 University of Copenhagen, Faculty of Health and Medical Sciences, Department of Immunology and Microbiology, Copenhagen, Denmark

**Keywords:** Predictive markers, Cardiovascular genetics, Risk factors

## Abstract

The AB0 blood group has been linked to ischaemic heart disease, stroke, and periodontal disease, while the Lewis blood group has been linked to ischaemic heart disease and obesity, all of which have been associated with periodontitis. AB0 or Lewis blood group phenotype may therefore constitute common hereditary components predisposing to these disorders. In this study, we investigated if blood group phenotype associated with periodontitis in a subpopulation consisting of 702 participants from a Danish cross-sectional cohort and, secondarily, attempted to confirm their association with hypertension, ischaemic heart disease, stroke, and obesity. No significant association between blood group phenotype and periodontitis was detected, nor were previously reported associations between blood group phenotype and hypertension, ischaemic heart disease, stroke, and obesity confirmed. This may, at least partly, be attributed to differences in study type, outcome definitions, cohort sizes, and population attributable factors. However, our results suggested a strong association between self-reported stroke and the Lewis (a−b−) phenotype (P = 0.0002, OR: 22.28; CI 95: 4.72–131.63).

## Introduction

The AB0 and Lewis blood group systems have both been linked to ischaemic heart disease (IHD)^1–4^. In addition, the AB0 blood group has been associated with stroke^5^, while the Lewis blood group has been associated with obesity^3^. All of these conditions have also been associated with periodontitis (PD)^6–8^, hence, AB0 and Lewis blood groups may constitute common hereditary components behind these disorders.

The AB0 blood group system comprises a major human alloantigen system, the phenotype of which is determined by three different allelic versions of the *AB0* gene located on chromosome 9, giving rise to the four possible AB0 blood groups; “A”, “B”, “AB”, and “0”. The *AB0* gene encodes a glycosyltransferase that catalyses the transfer of oligosaccharide moieties to the common H antigen, thereby forming the antigenic determinants of the A and B blood groups^9^.

The related Lewis blood group system comprises the two Lewis antigens, “Le^a^” and “Le^b^” each of which can either be present or absent, leading to four possible Lewis phenotypes: Le(a−b−), Le(a+ b−), Le(a−b+), and Le(a+ b+)^10^. Its basis is the fucosyltransferase 3 (FUT3)-encoding Lewis gene that controls formation of the Le^a^-antigen, and the fucosyltransferase 2 (FUT2)-encoding Secretor gene that controls formation of the Le^b^-antigen by converting Le^a^-antigen into Le^b^-antigen. FUT2 also controls the expression of AB0-antigens in the tissues; in the absence of an active FUT2 gene (non-secretors), no Le^b^ antigen will be formed, and AB0 antigens will not be expressed in the tissues^11^.

The phenotypic distribution of the two alloantigen systems varies tremendously with both geography and ethnicity suggesting that it has been subject to selection pressure. Indeed, blood group 0 appears to confer a relative resistance to severe malaria, while non-0 subjects appear to have reduced susceptibility to severe infections with *V. cholera* and *E. coli*^12–14^. Correspondingly, Lewis blood group phenotype has been associated with susceptibility to rotavirus and *Helicobacter pylori* infection^15,16^.

PD is a common disease characterized by chronic inflammation and destruction of the tooth-supporting tissues, ultimately leading to tooth loss^17^. PD can present late in life in a chronic slowly progressive form or, more rarely, earlier in life in an aggressive form with rapid tissue deterioration^18^. The causal and pathogenic differences behind these divergent forms of PD remain unclear, but a common denominator is destruction of the periodontal tissues, that is believed to be caused by inflammatory processes initiated by bacterial colonization of the tooth surfaces^19^. Approximately half of the adult population above 50 years of age suffer from PD to varying degrees^20^, but the exact prevalence is uncertain due to lack of consensus case criteria^21^.

The AB0 blood group phenotype has been associated with PD in several studies^22–27^, but to our knowledge no similar studies on Lewis blood group phenotype have yet been published.

In view of the proposed link between blood group phenotypes and ischemic heart disease, stroke, and obesity, and between these conditions and PD, we hypothesized that Lewis and AB0 blood groups constitute risk factors for PD as well and tested this hypothesis in a Danish cross-sectional cohort. The validity of these investigations rest on the assumptions of existing associations between blood group phenotypes and hypertension, ischaemic heart disease, stroke, and obesity, hence we attempted to confirm these associations as well.

## Results

### Demographics

The distribution of the study population exposures and covariables by PD case definitions, obesity, IHD, stroke, and hypertension is shown in Table 1.

**Table 1:** Distribution of study population exposure- and covariables by periodontitis case definitions, obesity, ischaemic heart disease, stroke, and hypertension. CAL-tertile; Clinical Attachment Level-tertile case criterium(REF). BMI; Body Mass Index (defined as weight (kg)/height squared (m2)), IHD; Ischaemic Heart Disease (self-reported), Stroke; self-reported stroke, Hypertension; self-reported or measured hypertension (diastolic pressure ≥ 100 mmHg or systolic pressure ≥ 150 mmHg).

	All	Severe periodontitis	CAL-tertile	Hypertension	IHD (self reported)	Stroke (self reported)	Obesity (BMI ≥ 30)
All (N (%))	702 (100)	112 (16)	197 (28)	275 (39)	19 (3)	16 (2)	92 (13)
Age (mean (SD))							
Years	55 (3)	56 (3)	55 (3)	55 (3)	55 (3)	57 (3)	55 (3)
Gender (N (%))							
Men	504 (72)	90 (18)	160 (32)	223 (44)	17 (3)	10 (2)	77 (15)
Women	198 (28)	22 (11)	37 (19)	52 (26)	2 (1)	6 (3)	15 (8)
Smoking (N (%))							
Never	254 (36)	17 (7)	32 (13)	87 (34)	4 (2)	6 (2)	36 (14)
Previous	288 (41)	43 (15)	85 (30)	124 (43)	3 (1)	7 (2)	37 (13)
Present	160 (23)	52 (33)	80 (50)	64 (40)	12 (8)	3 (2)	19 (12)
Alcohol, drinks per week (N (%))							
None	72 (10)	10 (14)	23 (32)	28 (39)	4 (6)	3 (4)	11 (15)
1–7 (women)/1–14 (men)	376 (54)	59 (16)	101 (27)	145 (39)	7 (2)	7 (2)	46 (12)
8–14 (women)/15–21 (men)	125 (18)	17 (14)	31 (25)	40 (32)	1 (1)	1 (1)	15 (12)
>14 (women)/>21 (men)	129 (18)	26 (20)	42 (33)	62 (48)	7 (5)	5 (4)	20 (16)
Diabetes (N (%))							
No (HbA1c <6.5%)	667 (95)	104 (16)	181 (27)	251 (38)	14 (2)	16 (2)	77 (12)
Yes (HbA1c≥6.5%)	35 (5)	8 (23)	16 (46)	24 (69)	5 (14)	0 (0)	15 (43)
Social Class (N (%))							
Social class I	71 (10)	11 (15)	15 (21)	26 (37)	0 (0)	2 (3)	8 (11)
Social class II	227 (32)	31 (14)	55 (24)	74 (33)	6 (3)	4 (2)	21 (9)
Social class III	187 (27)	28 (15)	56 (30)	78 (42)	5 (3)	4 (2)	25 (13)
Social class IV	123 (18)	22 (18)	40 (33)	50 (41)	0 (0)	3 (2)	21 (17)
Social class V	41 (6)	9 (22)	12 (29)	21 (51)	2 (5)	0 (0)	7 (17)
Transfer income	53 (8)	11 (21)	19 (36)	26 (49)	6 (11)	3 (6)	10 (19)
Body Mass Index (mean (SD))							
Weight/height^2^	26 (4)	27 (5)	27 (4)	27 (4)	27 (3)	26 (4)	33 (4)
AB0 bloodtype (N (%))							
0	271 (39)	47 (17)	78 (29)	101 (37)	6 (2)	6 (2)	35 (13)
A	308 (44)	46 (15)	77 (25)	123 (45)	9 (3)	7 (2)	40 (13)
B	97 (14)	11 (11)	34 (35)	42 (43)	4 (4)	3 (3)	15 (15)
AB	26 (4)	8 (31)	8 (31)	9 (35)	0 (0)	0 (0)	2 (8)
Lewis bloodtype (N (%))							
a−b−	93 (13)	18 (19)	29 (31)	30 (32)	0 (0)	9 (10)	10 (11)
a−b+	481 (69)	79 (16)	142 (30)	187 (39)	14 (3)	5 (1)	62 (13)
a + b−	127 (18)	15 (12)	26 (20)	58 (46)	5 (4)	2 (2)	20 (16)
a + b+	1 (0)	0 (0)	0 (0)	0 (0)	0 (0)	0 (0)	0 (0)
Clinical attachment level (mean (SD)), mm	2.39 (0.61)	3.29 (0.99)	3.04 (0.82)	2.47 (0.74)	2.76 (0.67)	2.31 (0.30)	2.47 (0.62)
Diastolic blood pressure (mean (SD)), mmHg	88 (10)	88 (9)	89 (10)	95 (9)	87 (10)	87 (9)	93 (9)
Systolic blood pressure (mean (SD)), mmHg	136 (17)	136 (17)	138 (17)	149 (17)	134 (11)	133 (14)	145 (17)
Cholesterol (mean (SD)), mmol/l	5.36 (0.82)	5.35 (0.90)	5.33 (0.87)	5.40 (0.84)	4.97 (0.85)	4.76 (0.71)	5.40 (0.81)
High sensitivity-CRP (mean (SD)), mg/l	1.92 (3.45)	2.30 (2.87)	2.18 (2.82)	2.52 (4.67)	1.62 (1.68)	2.37 (4.73)	4.57 (7.11)
Low-density lipoprotein (mean (SD)), mg/l	2.77 (0.72)	2.75 (0.74)	2.74 (0.80)	2.81 (0.74)	2.46 (0.79)	2.12 (0.79)	2.82 (0.72)
High-density lipoprotein (mean (SD)), mg/l	1.46 (0.38)	1.37 (0.36)	1.36 (0.36)	1.40 (0.36)	1.28 (0.32)	1.44 (0.26)	1.23 (0.31)
Triglycerides (mean (SD)), mg/l	1.80 (1.05)	2.08 (1.18)	2.06 (1.31)	2.05 (1.18)	2.12 (1.02)	1.87 (2.13)	2.61 (1.35)

The age of the participants ranged narrowly between 50 and 61 years, with men comprising the majority of the cohort (72%).

In the present cohort, according to the CAL-tertile definition, 197 (28%) participants were defined as periodontitis patients, while 112 (16%) were classified as having severe periodontitis. Severe periodontitis was significantly associated with IHD (P = 0.03, OR: 4.15; CI 95: 1.17–15.67) but not with obesity, stroke or hypertension (data not shown). Periodontitis in general was not significantly associated to any of the conditions studied (data not shown).

The average BMI among participants ranged between 17 and 58 with a mean of 26, and 92 participants (13%) were classified as obese with a BMI of at least 30. Obesity was significantly associated with diabetes, social class, hypertension, CRP-level, triglyceride level, and inversely related to high density lipoprotein level (Table 2).

**Table 2:** Multivariable logistic regression analyses with periodontitis case definitions, obesity, ischemic heart disease, stroke, and hypertension as outcomes.

Risk factor (OR (CI 95%))	Severe periodontitis	CAL-tertile	Hypertension	IHD (self reported)	Stroke (self reported)	Obesity (BMI ≥ 30)
Blood group A positive	1.26 (0.73–2.16)	0.66 (0.42–1.02)	1.08 (0.76–1.53)	0.70 (0.19–2.37)	0.35 (0.06–1.61)	0.85 (0.48–1.47)
Blood group B positive	0.70 (0.35–1.35)	1.25 (0.74–2.10)	0.98 (0.63–1.53)	0.86 (0.19–3.24)	0.53 (0.06–2.98)	0.72 (0.35–1.42)
Lewis negative (a−b−)	1.40 (0.67–2.86)	1.42 (0.75–2.64)	0.69 (0.4–1.14)	N.A.	**22.28 (4.72–131.63)**	0.77 (0.32–1.70)
Age (decades)	**2.77 (1.13–6.86)**	2.01 (0.98–4.12)	**2.69 (1.53–4.78)**	0.95 (0.09–10.02)	**37.04 (4.94–446.68)**	1.71 (0.68–4.29)
Gender (Men)	0.69 (0.34–1.42)	1.39 (0.78–2.49)	1.29 (0.82–2.04)	2.58 (0.38–26.56)	0.63 (0.15–3.02)	0.90 (0.43–1.99)
Previous smoking	1.50 (0.77–3.00)	**2.60 (1.57–4.40)**	1.31 (0.89–1.92)	0.38 (0.06–2.08)	1.87 (0.45–9.01)	0.68 (0.38–1.22)
Present smoking	**2.99 (1.47–6.23)**	**4.92 (2.76–8.92)**	1.06 (0.65–1.72)	2.87 (0.65–14.77)	1.52 (0.19–11.11)	**0.37 (0.16–0.81)**
Alcohol abstaining	0.43 (0.16–1.06)	1.22 (0.60–2.41)	0.95 (0.52–1.7)	1.39 (0.25–6.66)	3.60 (0.47–23.83)	0.99 (0.39–2.33)
Drinks per week: 8–14 (women)/15–21 (men)	0.76 (0.36–1.53)	1.04 (0.58–1.83)	0.8 (0.5–1.28)	0.56 (0.03–4.00)	0.40 (0.02–3.03)	1.16 (0.54–2.36)
Drinks per week: >14 (women)/>21 (men)	1.08 (0.54–2.09)	1.04 (0.58–1.82)	1.27 (0.81–2)	3.47 (0.81–15.83)	2.50 (0.52–11.51)	1.21 (0.59–2.45)
Social class I	1.90 (0.73–4.75)	0.97 (0.42–2.12)	1.43 (0.78–2.61)	N.A.	1.54 (0.13–12.83)	1.95 (0.70–5.10)
Social class III	0.89 (0.45–1.74)	1.39 (0.82–2.38)	1.43 (0.93–2.2)	0.99 (0.23–4.20)	0.89 (0.14–5.53)	1.73 (0.86–3.55)
Social class IV	1.06 (0.50–2.22)	1.47 (0.80–2.67)	1.34 (0.82–2.19)	N.A.	0.78 (0.09–5.33)	**2.39 (1.10–5.20)**
Social class V	1.70 (0.56–4.83)	1.03 (0.39–2.59)	**2.37 (1.14–4.99)**	3.82 (0.43–27.12)	N.A.	2.50 (0.82–7.06)
Transfer income	1.17 (0.41–3.18)	1.09 (0.46–2.48)	1.66 (0.83–3.31)	**5.65 (1.18–28.90)**	5.84 (0.57–59.96)	1.58 (0.53–4.36)
Diabetes (HbA1c ≥ 6.5%)	0.67 (0.21–1.99)	1.58 (0.62–3.90)	2.07 (0.92–4.85)	**7.09 (1.31–40.35)**	N.A.	**3.36 (1.38–8.10)**
Cholesterol (mmol/l)	1.27 (0.63–2.55)	0.99 (0.56–1.73)	0.94 (0.6–1.47)	0.90 (0.15–4.49)	0.76 (0.10–5.04)	1.02 (0.49–2.08)
High sensitivity-C-reactive protein (mg/l)	1.01 (0.92–1.07)	0.99 (0.92–1.05)	**1.07 (1.01–1.14)**	0.85 (0.57–1.07)	0.91 (0.73–1.08)	**1.22 (1.13–1.31)**
Low density-lipoprotein (mmol/l)	0.72 (0.34–1.51)	0.92 (0.50–1.68)	1.1 (0.68–1.79)	0.68 (0.12–3.91)	0.20 (0.02–1.61)	1.16 (0.53–2.53)
High density-lipoprotein (mmol/l)	0.64 (0.24–1.67)	0.62 (0.28–1.38)	0.93 (0.49–1.73)	0.35 (0.03–4.22)	1.23 (0.10–11.52)	**0.28 (0.09–0.84)**
Triglyceride (mmol/l)	1.04 (0.78–1.39)	1.05 (0.82–1.34)	1.22 (0.99–1.51)	0.75 (0.35–1.55)	1.22 (0.58–2.62)	**1.46 (1.10–1.95)**

CAL-tertile; Clinical Attachment Level-tertile case criterium (REF). BMI; Body Mass Index (defined as weight (kg)/height squared (m2)), IHD; Ischaemic Heart Disease; Hypertension; self-reported or measured hypertension (diastolic pressure ≥ 100 mmHg or systolic pressure ≥ 150 mmHg), Actual ages were divided by ten (decades). OR; Odds ratio, CI 95%; 95% confidence interval. Statistically significant associations are marked in bold (P ≤ 0.05).

Nineteen (3%) of the participants had experienced myocardial infarction or angina pectoris, 16 (2%) had experienced a stroke, and 275 (39%) had hypertension, either self-reported or according to blood pressure measurements, or use of anti-hypertensive medication. IHD was significantly associated with social class and diabetes, and stroke was strongly associated with age, while hypertension was associated with age, social class, obesity and CRP-levels (Table 2).

### Lewis and AB0 blood group associations

No significant associations were observed between AB0 or Lewis blood groups and either of the included definitions of PD.

Although only 16 among the 702 participants had experienced a stroke, a highly significant association to the Lewis(a−b−) blood group was observed for this group (P = 0.0002, OR: 22.28; CI 95: 4.72–131.63 - Table 2). Among stroke victims, 56% (9 of the 16 participants) carried the Lewis(a−b−) blood group, compared to only 14% (84 of 686) Lewis(a−b−) among the remaining participants. In contrast, IHD was not reported by any of the Lewis(a−b−) participants.

No significant associations were observed between Lewis blood group and hypertension, IHD, or obesity, nor were AB0 blood groups significantly associated with any of the investigated parameters (Table 2).

## Discussion

We investigated the association of Lewis and AB0 blood groups with PD and sought to confirm previously reported associations between blood group phenotype and hypertension, IHD, stroke, and obesity in a subpopulation of the CAMB-cohort consisting of 702 participants. The AB0 blood group has been linked to IHD^1^, stroke^5^, and periodontal disease^22–27^, while the Lewis blood group has been linked to IHD^2^ and obesity^3^, all of which have been associated with PD^6–8,28,29^. We were not able to confirm any of those relationships, however. Notably, associations with PD remained non-significant, even though we included two accepted but very different PD definitions with widely ranging frequencies (28% vs. 16%, respectively).

By contrast, we observed a strong association between the Lewis(a−b−) phenotype and self-reported (non-fatal) stroke incidence: OR (CI 95%) = 22.28 (4.72–131.63). To our knowledge, this has not previously been reported, although IHD and ischemic stroke share risk factors and have similar pathophysiologies^30^.

Besides age and Lewis blood group, none of the included covariables were significantly associated with stroke (Table 2), so it does not seem likely that factors that may be associated with ischaemic conditions (e.g. blood pressure, diabetes, triglyceride and cholesterol levels) can explain the observed association between Lewis blood group and stroke.

Conversely, the relationship between stroke and rheological parameters, particularly plasma viscosity, has been shown by others to be at least as strong as its relationship to conventional risk factors such as smoking, blood pressure, and cholesterol levels^31^. Additionally, rheological parameters, including erythrocyte sedimentation rate, red blood cell aggregation, and plasma viscosity, have been shown to be markedly aggravated in Le-negative individuals^32^. This supports our finding of a significantly increased occurrence of stroke in Le-negative individuals.

Stroke incidence was self-reported, so it cannot be determined whether the stroke(s) were ischaemic or haemorrhagic. However, ischaemic stroke is by far (>82%) the more common form^33^, and this fits well with previously reported associations between the Lewis(a−b−) phenotype and ischaemic disease^2^. Although self-reported diagnoses may be associated with considerable uncertainty depending on survey method, participant characteristics, and reference standard used^34^, acceptable error rates have been observed in health surveys comparable to ours^35^.

It should be noted that this study was cross-sectional without the possibility of following participants prospectively. Hence, only non-fatal IHD and stroke reports were included. In another Danish cross-sectional study, the rate of fatal strokes among all strokes observed was estimated to be 19%^36^. Interestingly, there was no overlap between participants reporting a history of stroke and those reporting a history of IHD in this study. Based on the observed Lewis(a−b−) proportion of the cohort, at least 2–3 Lewis(a−b−) participants would have been expected in the IHD group. A possible explanation for this observation could be that there is an increased incidence of fatal IHD among Lewis(a−b−) subjects, as has been reported previously^2^. Given the cross-sectional nature of our study in combination with the relatively low number of participants reporting IHD, it seems likely that IHD-events among potential Lewis(a−b−) participants could have been fatal. Moreover, cerebral ischemia has previously been associated with asymptomatic IHD and increased risks of fatal IHD following stroke has been reported^37^.

Unexpectedly, we did not observe significant associations between age and IHD, nor between age and obesity in this cohort. These observations may be attributed to the skewed and very narrowly ranged age distribution within the cohort, possibly in combination with a pronounced overrepresentation of male participants.

It should also be taken into account that PD, obesity, IHD, stroke, and hypertension are all complex conditions that are under the influence of numerous parameters such as medication and general lifestyle factors like diet and excercise.

In conclusion, the results of the present study do not support the hypothesis of a role for Lewis or AB0 blood groups in PD, nor were previously reported associations between blood group phenotype and hypertension, ischaemic heart disease, stroke, and obesity confirmed. This may, at least partly, be attributable to differences in study type, outcome definitions, cohort sizes, and population attributable factors. As a secondary observation, however, our results indicate a strong association between the Lewis(a−b−) phenotype and self-reported (non-fatal) stroke incidence, which warrants further investigations.

## Materials and Methods

### Study population

Study participants comprised a subpopulation from the Copenhagen Aging and Midlife Biobank (CAMB) whose oral health was assessed during the establishment of CAMB in 2009–2011^38^. Briefly, CAMB is a merger of three established cohorts: The Metropolit Cohort (10,171 men born in Copenhagen in 1953, age at follow-up 56–58), The Copenhagen Perinatal Cohort (8,102 men and women born at the National University Hospital in Copenhagen in 1959–1961, age at follow-up 49–52), and the Danish Longitudinal Study on Work, Unemployment, and Health (11,082 men and women, born 1949 and 1959, constituting a random sample of the Danish population in 1999, age at follow-up 50–53 and 60–63. 17,937 eligible persons from the three established cohorts living in the Eastern parts of Denmark were invited to participate and of these, 5,575 came to a study clinic for further testing and blood sampling. A total of 1,517 participants from this group took part in The CAMB Oral Health Study as described previously^39^. Of these, 702 had complete registrations for all of the selected exposures and covariables as well as blood samples forming the basis of the present study.

### Covariables

Of the 702 participants with complete data, 504 (72%) were men. Age at the time of inclusion ranged from 50 to 61 years, with a mean of 55. Smoking status was included as three categories (never, previous, and present smoker). Weekly alcohol consumption was included using the categorization employed by the Danish Health Authorities: 0 drinks per week, 1–7 drinks per week (women)/1–14 drinks per week (men), 8–14 drinks per week (women)/15–21 drinks per week (men), and >14 drinks per week (women)/>21 drinks per week (men), based on a standard Danish drink definition equalling 12 g of pure alcohol. Diabetes diagnosis was based on increased haemoglobin A1c levels (≥6.5%), a self-reported diabetes diagnosis, or both. Social class was classified by occupation and coded into Social Classes I–VIII, according to the standards of the Danish Occupational Social Class classification^40^. According to this classification, Social Classes I–V encompass economically active individuals ranging from professional occupation in Social Class I to unskilled occupation in Social Class V. Social classes VI–VIII represent people on transfer income, students, homemakers, and those with no information on social class. In the present study, we combined class VI–VIII into one. Body Mass Index (BMI) (defined as weight (kg)/height squared (m2)) was included as a continuous variable. Blood pressure was recorded in mm Hg and reported as the mean of two subsequent measurements. Cholesterol, lipoprotein and triglyceride levels were included as continuous variables (Table 1).

### Case definitions

Oral examinations were carried out by two trained dental hygienists who followed a standardized examination protocol performed in fully equipped, stationary dental clinics that included air syringes, suction, and overhead lights. Each examiner used a front-surface dental mirror, caries explorer and periodontal probe. Re-examinations were not performed due to time constraints. Data were recorded on paper examination forms by trained recorders and subsequently entered into an electronic database^39^.

Large discrepancies in e.g. PD frequency may arise depending on the choice of PD case definition, of which several exist. To accommodate this, we included two definitions assumed to broadly represent most of the variation between different PD case definitions applied in previous publications; The ‘severe PD’-definition, comprising individuals with at least two interproximal sites on different teeth with a minimum CAL of six millimeters, as well as at least one interproximal site with a pocket depth of five millimeters or more^41^, and the ‘CAL-tertile’-definition, comprising individuals with a minimum CAL of three millimeters on at least one third of their measurement sites^42^.

Obesity was defined as a BMI of at least 30 kg/m2.

Ischaemic heart disease (IHD) was included as a binary variable defined as a self-reported previous incidence of myocardial infarction or angina pectoris.

Stroke was included as a binary variable and defined as a self-reported previous incidence of stroke.

Hypertension was included as a binary variable defined by self-reported hypertension, self-reported use of anti-hypertensive medicine, a measured systolic blood pressure of at least 150 mm Hg, or a measured diastolic blood pressure of at least 100 mm Hg.

### Lewis and AB0 blood group typing

Lewis blood group typing was performed as described by the vendor (Biotest AG, Dreieich, Germany). Briefly, 100 microliters of fresh anti-coagulated venous blood were washed in a clear Falcon tube (VWR International A/S, Soeborg, Denmark) by the addition of 1 ml of Dulbecco’s phosphate buffered saline (PBS) (Life Technologies Europe BV, Naerum, Denmark) followed by a brief centrifugation at 900 g to pellet blood cells. The supernatant was discarded, and the procedure was repeated until the supernatant remained clear. One microliter of the washed blood cells was transferred to each of two clear Falcon tubes containing 39 microliters of PBS, and 40 microliters of either Seraclone anti-Lea or Seraclone anti-Leb (Biotest AG, Dreieich, Germany) were added to the suspensions. The tubes were incubated for 15 minutes at ambient temperature and centrifuged briefly at 800 g. Finally, agglutination was assessed visually after gently loosening the pellet.

AB0 blood group typing was performed as described by the vendor (Biotest AG, Dreieich, Germany). Briefly, 100 microliter aliquots of test serum were placed in clear Falcon tubes and added 50 microliters of either Biotestcell antigen A1, A2, B, or 0. After careful mixing, samples were incubated 15–30 minutes at ambient temperature and centrifuged at 150 g for 2 minutes. Pellets were subsequently loosened and assessed for agglutination.

All blood group typings were performed blinded and by the same technician.

### Ethical approval and informed consent

The study was approved by The Ethics Committee for The Capital Region of Denmark (#H-A-2008-126). The study was carried out in accordance with relevant guidelines and regulations. Informed consent was obtained from all participants.

### Statistical analyses

Numerical variables were expressed as means with standard deviations (SD). Categorical variables were expressed as numbers (n) with percentages (%). Covariable-adjusted logistic regression analyses were applied to assess cross-sectional relationships between Lewis- and AB0-blood type and PD, hypertension, IHD, stroke, and obesity. Analysis results were expressed as odds ratios (OR) with 95% confidence intervals (CI 95%). Two-tailed P < 0.05 was considered statistically significant. Reference categories were chosen by biological relevance as well as population incidence. All analyses were performed in RStudio Version 1.0.153 (RStudio Inc., Boston, MA, USA) using R version 3.4.2 (R Foundation for Statistical Computing, Vienna, Austria).

## Data Availability

The data that support the findings of this study are available from The Copenhagen Aging and Midlife Biobank, but restrictions apply to the availability of these data, which were used under license for the current study, and so are not publicly available. However, data are available from the authors upon reasonable request and with permission of The Copenhagen Aging and Midlife Biobank steering committee.
